# Mechanistic Insights Into Trehalose-Mediated Cold Stress Tolerance in Rapeseed (*Brassica napus* L.) Seedlings

**DOI:** 10.3389/fpls.2022.857980

**Published:** 2022-03-10

**Authors:** Ali Raza, Wei Su, Ziqi Jia, Dan Luo, Yi Zhang, Ang Gao, Muhammad Azhar Hussain, Sundas Saher Mehmood, Yong Cheng, Yan Lv, Xiling Zou

**Affiliations:** Key Laboratory of Biology and Genetic Improvement of Oil Crops, Oil Crops Research Institute, Chinese Academy of Agricultural Sciences, Wuhan, China

**Keywords:** abiotic stress, antioxidant defense system, gene expression, marker genes, osmoprotectants, trehalose metabolism, chilling stress

## Abstract

Cold stress (CS) severely affects several physiological, biochemical, and molecular mechanisms and limits the growth and production of rapeseed (*Brassica napus* L.). Trehalose (Tre) acts as a growth modulator, which is extensively used to improve the tolerance to multiple plant stresses. Further, Tre also serves as an external force in inducing plant signaling molecules, regulating the expression of stress-responsive genes, and enhancing the CS tolerance in plants. Nevertheless, the importance of exogenous Tre in improving the CS tolerance in rapeseed is still unclear. Therefore, the current study was designed to get mechanistic insights into Tre-mediated CS tolerance in rapeseed seedlings. To explore the Tre role, we designed four treatments [control (CK), CK + 20 mM L^–1^ Tre, Cold, and Cold + 20 mM L^–1^ Tre] and three CS conditions (4, 0, and −4°C). The results showed that Tre treatments significantly mitigated the adverse effects of CS on the seedlings and increased the survival rate of Tre-treated seedlings under CS conditions. The exogenous Tre dramatically increased the contents of osmoprotectants, including the soluble sugar (SS), soluble protein (SP), and proline (Pro), and the activities of antioxidant enzymes, such as catalase (CAT), peroxidase (POD), superoxide dismutase (SOD), and ascorbate peroxidase (APX) were also increased under CS conditions. Additionally, Tre decreased the malondialdehyde (MDA) contents to protect the rapeseed seedlings. Moreover, Tre also remarkably augmented the expression levels of antioxidant genes (*CAT12, POD34*, and *FSD7*), CS-responsive marker genes (*CBF1, CBF2, CBF4, COR6.6, COR15, COR25, COL1*, and *KIN1*), and Tre-biosynthesis genes (*TPS4, TPS8*, and *TPS9*). Briefly, exogenous Tre not only regulates the antioxidant and osmotic balance, but it also significantly participates in Tre metabolism and signaling network to improve the CS tolerance in rapeseed. Thus, Tre-induced supervisory connections between physiological or/and biochemical attributes provide information to dissect the mechanisms of Tre-mediated CS tolerance.

## Introduction

Plants are constantly confronted by the changing climate, which leads to several environmental stresses. Amongst them, cold stress (CS) [(chilling (0–15°C) and freezing temperature (<0°C)] is a main abiotic factor that influences the productivity of the plants, restricts the geographical circulation, and lessens crop yields (He et al., 2021; Mehmood et al., 2021). Consequently, CS leads to alterations in the physiological, biochemical, and molecular mechanisms, as reviewed by Raza et al. (2021c) and Zahra et al. (2021). Short-term CS enhances the levels of reactive oxygen species (ROS) (He et al., 2021), triggers mitogen-activated protein kinase cascades (MAPKs/MPKs), activates down-stream C-repeat binding factor (CBF)-dependent or CBF-independent pathways (Lohani et al., 2020; Raza et al., 2021a), and eventually adjusts the expression levels of CS-responsive genes (Ding et al., 2019; Lei et al., 2019; He et al., 2021). On the contrary, long-term CS triggers the overproduction of ROS and redox imbalance (Ding et al., 2019; Hasanuzzaman et al., 2020; Mehmood et al., 2021). In contrast, the ROS accumulation damaged large molecules, including DNA, proteins, lipids, cell structure and membrane, and the physio-biochemical and metabolic processes (Hasanuzzaman et al., 2020). Subsequently, CS reduces the cell membrane fluidity, photosynthesis, crop productivity, and even leads to cell death (Ding et al., 2019).

During the previous two decades, several elements, such as messenger molecules (ROS calcium, and phytohormones), protein kinases and phosphatases (MAPKs/MPKs, CDPKs, CBLs, CIPKs, etc.), and transcription factors (CBF, COR, DREB, etc.) have been identified in the CS signaling pathways (Wang et al., 2017; Shi et al., 2018; Ding et al., 2019; Raza et al., 2021a). So far, ICE-CBF-COR signaling pathways have been best characterized under CS conditions. Mainly, *CBF* genes can quickly be induced by CS, and they play substantial roles in the cold acclimation phenomenon (Wang et al., 2017; Shi et al., 2018), whereas *COR* genes are mainly regulated by CS, including *COR, KIN, COL*, etc., (Shi et al., 2018). Interestingly, these genes encode cryoprotective and osmolyte proteins to protect plants from freezing damage (Shi et al., 2018). Moreover, *CBFs* can directly attach with the *COR* genes promoters and induce their expression levels, thus, improving the CS tolerance in plants (Liu et al., 1998).

In the recent past, several plant growth regulators have been extensively applied to improve the stress tolerance in crop plants (Mubarik et al., 2021; Raza et al., 2021d; Sabagh et al., 2021). Among them, trehalose (Tre) is a non-reducing disaccharide sugar that possesses two glucose molecules (Wingler, 2002). The Tre mainly exists in different organisms, including yeasts, bacteria, invertebrates, and in a smaller amount in plants, that alleviate osmoprotectant molecules and are easily absorbed by the plants (Wingler, 2002; Kosar et al., 2019). The endogenous Tre content was found to be very low in plants, such as rice and tobacco (Kretovich, 1980); nevertheless, it is greatly induced by numerous abiotic stresses, including CS (Kosar et al., 2019; Joshi et al., 2020; Zulfiqar et al., 2021a). A study has been concentrated on the clarification of Tre metabolism, especially in transgenic crops with stress tolerance (Schwarz and Van Dijck, 2017). A low amount of Tre is not merely because of the activity of Tre but also owes to strong modulation of trehalose-6-phosphate synthase (*TPS*) and trehalose-6-phosphate phosphatase (*TPP*) gene expression and enzyme activities (Delorge et al., 2014). Interestingly, Tre not only contributes to plant metabolisms but also contributes to signaling mechanisms (Paul et al., 2008). Furthermore, the exogenously sprayed Tre can quickly accumulate and be moved by the roots and the leaf tissues, showing significant roles as osmoprotectants (Luo et al., 2010). Previously, it has been documented that Tre regulates different osmotic substances in various plant species under stress conditions, e.g., osmotic pressure, chillness, drought, and heat stress (Luo et al., 2008; Liu et al., 2020; Zulfiqar et al., 2021a). Recently, Williams et al. (2015) described that Tre was extensively distinguished in CS-tolerant crop plants, such as *Tripogon loliiformis*, signifying that Tre was involved in developing CS tolerance in diverse plant species.

In the recent years, limited studies have been carried out using exogenous Tre to enhance CS tolerance in different plant species, such as tomato (*Solanum lycopersicum* L.) (Liu et al., 2020), rice (*Oryza sativa* L.) (Fu et al., 2020), wheat (*Tritum aestivum* L.) (Liang et al., 2021), melon (*Cucumis melo* L.) (Liu et al., 2021), etc., and the mechanisms mediating Tre effects have not been fully discovered. Most importantly, the effect of exogenous Tre in improving the CS tolerance in rapeseed (*Brassica napus* L.) is still unclear. Rapeseed is the third-leading oilseed crop in China that is frequently exposed to CS, mainly in the winter (He et al., 2021; Li et al., 2021; Raza, 2021; Su et al., 2021a). The CS significantly impairs rapeseed productivity and ultimately reduces biomass and seed harvest (Raza, 2021). Hence, the current experiment was designed to elucidate the positive role of exogenous Tre in improving the CS tolerance in rapeseed. To get insights into Tre-mediated CS tolerance, we examined the physiological and biochemical indices in the seedling treated with exogenous Tre. Furthermore, we also analyzed the expression levels of CS-responsive antioxidants and Tre-biosynthesis genes to identify the key effects of Tre application at the molecular level, which could be acting as an expressive approach for unraveling the mechanistic insights of Tre in enhancing the CS tolerance in rapeseed.

## Materials and Methods

### Plant Material and Growth Conditions

The seeds of a widely cultivated rapeseed variety, “ZhongShang11 (ZS11)” (Sun et al., 2017), were supplied by the Oil Crops Research Institute, the Chinese Academy of Agricultural Sciences (CAAS), Wuhan, China. The vigorous seeds of ZS11 were grown on moist filter paper on petri dishes in a chamber (25°C day/night and 16 h/8 h light/dark cycle). After 7 days, the seedlings were moved to pots (10 cm, four plants pot^–1^) containing a mixture of vermiculite and nutrient soil (2:1). The 21-day-old (four-leaf stage) seedlings were exposed to CS treatments.

### Treatments and Sample Harvesting

The research was performed in a completely randomized design, including three biological replications. We designed four treatments [control (CK), CK + Tre, Cold, and Cold + Tre] and three stress conditions (4, 0, and −4°C) to investigate the effects of exogenous Tre (CAS#99-20-7) on rapeseed that has CS tolerance. The Tre was purchased from Yuanye Bio-Tech Co., Ltd., (Shanghai, China). Before CS treatments, the whole plants were pretreated with 20 mM L^–1^ of Tre solution and distilled water at room temperature [25°C/18°C (day/night)]. The Tre concentration (20 mM L^–1^) was selected based on previous studies (Ibrahim and Abdellatif, 2016; Samadi et al., 2019; Liu et al., 2020). The selected pots for Tre treatment were sprayed with 10 mL of Tre solution, and the CK seedlings were sprayed with 10 mL of distilled water. Every treatment was replicated three-times (three pots/replication) and arbitrarily organized. After 24 h, half of the seedlings were exposed to CS (4°C for 24 h, 0°C for 12 h, and −4°C for 6 h). The CK samples were kept at room temperature (25°C) with the corresponding treatment time. After CS treatments, the green leaves were harvested and immediately preserved in liquid nitrogen and kept at −80°C until the next analysis.

### Measurements of Physiological and Biochemical Attributes

To analyze the physiological and biochemical alterations in rapeseed under CS, the contents of soluble sugar (SS), soluble protein (SP), proline (Pro), malondialdehyde (MDA), and hydrogen peroxide (H_2_O_2_) were measured using commercial kits following the manufacturer’s guidelines. Similarly, the antioxidant defense enzymes activities, including catalase (CAT, EC 1.11.1.6), superoxide dismutase (SOD, EC 1.15.1.1), peroxidase (POD, EC 1.11.1.7), and ascorbate peroxidase (APX, EC 1.11.1.11), were measured using commercial kits following the manufacturer’s guidelines. The kits for SS (G0501W), SP (G0418W), Pro (G0111W), MDA (G0109F), H_2_O_2_ (G0112F), CAT (G0105F), SOD (G0101F), POD (G0107F), and APX (G0203F) were bought from Suzhou Grace Biotechnology Co., Ltd., (Suzhou, Jiangsu, China). The detailed protocol manuals are freely available at the company profile^1^ and can be extracted using the above-mentioned kit IDs. All the parameters were measured using three biological replications and a spectrophotometer microplate reader (Epoch, BioTek, Instruments, Inc., Winooski, VT, United States).

### Gene Expression Analysis

To boost our understanding of Tre-mediated CS tolerance in rapeseed seedlings, we evaluated the expression levels of antioxidant genes, stress-related marker genes, and genes involved in Tre biosynthesis/metabolism (Table 1). Antioxidant and Tre genes were identified and randomly selected using keywords in the rapeseed genome^2^. Total RNA was extracted using TransZol Up Plus RNA Kit (TransGen Biotechnology, Beijing, China) following the manufacturer’s guidelines. The RNA was treated and purified with TransScript One-Step gDNA Removal to eradicate the gDNA impurities. According to the manufacturer’s guidelines, the cDNA was harmonized utilizing a complementary DNA (cDNA) Synthesis SuperMix kit (TransGen Biotechnology, Beijing, China). The quantitative real-time-PCR (qRT-PCR) reaction was accomplished with an ABI StepOne real-time fluorescence quantitative PCR instrument (Applied Biosystems, CA, United States) following the reported method (Raza et al., 2021b). Briefly, the *BnACTIN* gene was used as an internal control. The qRT-PCR reaction was executed as follows: at 94°C for 10 min, followed by 40 cycles of 94°C for 15 s, 60°C for 30 s, and 72°C for 10 s. Gene expression analysis was carried out with three technical replications. The information of genes and primers used for qRT-PCR analysis are shown in Table 1.

**TABLE 1:** A list of primers used for the gene expression analysis.

Gene name	Gene ID/Accession number	Forward	Reverse
CBF1	BnaC07g39680D	CACCCAGTTTACAGAGGAGTTCG	ATCTCGGCTGTTAGGAAAGTACC
CBF2	BnaA03g13620D	TCTGAAATGTTGGGCTCCGA	CGCGTCTCCCGAAACTTCTT
CBF4	BnaA10g07630D	TTTCTCAGACTCGTTCCTCTCG	CTCCCTGCTCGTTTCTTCG
COR15	U14665.1	TCTCATTGGGATTGGTTCTTCTTT	ATGTTGCCGTCACCTTTATCG
COR25	HM187577.1	GGTCACAGCGAAAAACCAGAT	TCTTGGCGTGATAACCTGGAA
COR6.6	BnaA02g02910D	GGAGAAGGGTAATGTGCTGATGG	GCTACTTGTTCATGCCGGTCTT
COL1	BnaA02g02840D	CTTCTTGGTTGTTGCCTAGTTC	CATATGACATCCTGAGGACACA
KIN1	BnaAnng37980D	TCGTGCTGAGGAGAAGGGTA	CCGCCTCCGTTATCTTCTGT
CAT12	BnaC08T0260800ZS	AGCTTGCTTTCAACCCTGGT	CATTCACAGGCAGCTGGAGA
FSD7	BnaC03T0164000ZS	TTGATGCTCTAGAACCGCATAT	GATAGACTCCCAGAAGAACTCG
POD34	BnaC08g20820D	CGTGCTGATAACGTGTTAAGATTTG	CTCCCTTTGACTCCACCTTGT
TSP4	BnaA03g43320D	TTGTGGATTATGATGGGACGAT	AAACGATATTCTTGGGGTCACT
TSP8	BnaA07g24830D	CTAGCTGGACCTTCTCTCTAGA	GTCTTCTTTTAAGCAGCCTACG
TSP9	BnaA07g34230D	GACGTGATACAACACATGAAGG	GCGTTCTCCTCAAGAAACTTTT

### Statistical Data Analysis

The data were examined using GraphPad Prism Ver. 9 (Swift, 1997). The statistical significance was verified with a two-way ANOVA and Tukey’s test at a significant level of ^****^P < 0.0001, ^***^P ≤ 0.001, ^**^P ≤ 0.01, *P ≤ 0.05. Pearson’s correlation analysis was performed using the mcor function, and corrplots were prepared using corrplot package Ver. 0.89 (Wei et al., 2017) in RStudio (Allaire, 2012). Principal component analysis (PCA) was performed using fviz-pca function of the factoextra R package Ver. 1.0.7 (Kassambara and Mundt, 2020) in RStudio. All other graphs were prepared in GraphPad Prism.

## Results

### Exogenous Tre Improves the Survival Rate of Cold-Stressed Rapeseed Seedlings

**FIGURE 1:**
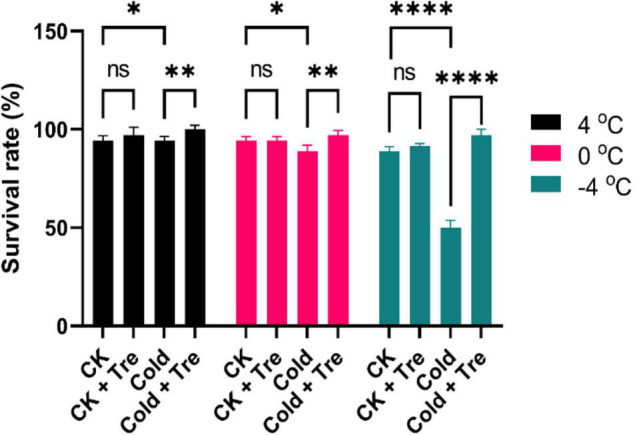
Impact of Tre on the survival rate of rapeseed seedlings under four treatments [control (CK), CK + Tre, Cold, and Cold + Tre] and three cold stress conditions (4, 0, and –4°C). Tre indicates the application of 20 mM L^– 1^ Tre. Data are expressed as the mean (± SD) from three biological replications for each sample and treatment. Asterisks show significant levels at ^****^P < 0.0001, ^**^P ≤ 0.01, *P ≤ 0.05, and ns means non-significant.

Cold stress (either freezing or/and chilling temperature) significantly impair the productivity of the rapeseed. Upon exposure to CS (4, 0, and −4°C), the rapeseed seedlings experienced a damaging phenomenon. Thus, the rapeseed seedlings were pretreated with Tre to evaluate their survival rate under CS conditions. Exogenous Tre significantly improved the survival rate compared to CK and cold-stressed seedlings, except for the chilling stress (0°C). For instance, under CS conditions, the Tre treatment showed a 100% survival rate at 4°C, and 97% survival rate at 0 and −4°C, which are significantly higher than CK seedlings without Tre application (Figure 1). Initially, the CS reduced the seedling growth, whereas the exogenous Tre significantly alleviated the survival rate of the rapeseed seedlings under freezing conditions (−4°C). Therefore, Tre pretreatment could be considered as a fascinating approach in mitigating the adverse effect of CS on rapeseed productivity.

### Impact of Tre on the Physiological and Biochemical Indices

#### Tre Promotes the Levels of Osmoprotectant Substances in Cold-Stressed Rapeseed Seedlings

**FIGURE 2:**
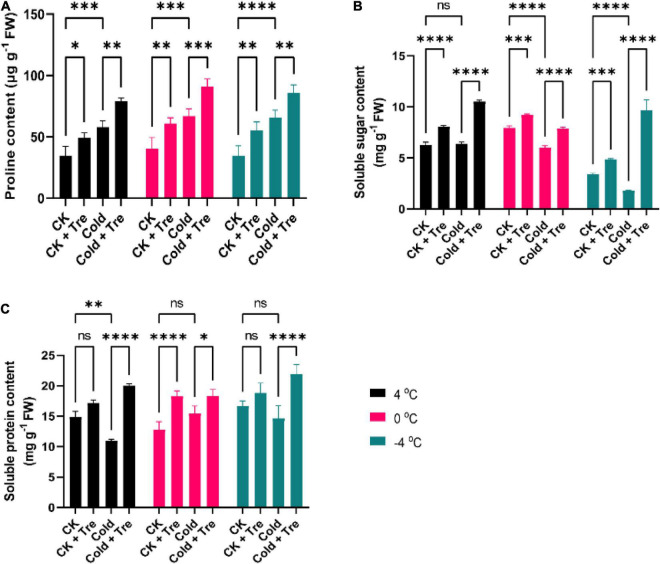
Impact of Tre on the levels of osmoprotectant substances in rapeseed seedling under cold stress conditions. **(A)** Proline contents, **(B)** soluble sugar contents, and **(C)** soluble protein contents under four treatments [control (CK), CK + Tre, Cold, and Cold + Tre] and three stress conditions (4, 0, and –4°C). Tre indicates the application of 20 mM L^– 1^ of Tre. Data are expressed as the mean (± SD) from three biological replications for each sample and treatment. Asterisks show significant levels at ^****^P < 0.0001, ^***^P ≤ 0.001, ^**^P ≤ 0.01, *P ≤ 0.05, and ns means non-significant.

Cold stress increased the Pro contents in the leaves of rapeseed seedlings. Cold stress led to increased Pro content by 167% at 4°C, 164% at 0°C, and 189% at −4°C, compared to CK (Figure 2A). Under normal conditions, the exogenous Tre improves the Pro contents by 141, 150, and 159% compared to the CK seedlings treated with water (Figure 2A). Under CS conditions, the application of Tre significantly increased the Pro contents by 227, 224, and 248% at 4, 0, and −4°C compared to CK, respectively (Figure 2A). Likewise, the CS decreased the SS contents by 75% at 0°C and 52% at −4°C compared to CK (Figure 2B). However, the application of Tre increased the SS contents by 128, 115, and 141% at 4, 0, and −4°C compared to CK, respectively (Figure 2B). In addition, under CS conditions, the SS contents of seedlings with Tre application were significantly increased by 167% at 4°C, and 281% at −4°C compared to CK. Additionally, CS reduced the SP contents at 4°C (by 73%) and −4°C (by 87%) compared to CK (Figure 2C). Under normal conditions, with Tre application, the SP contents were remarkably increased by 115, 142, and 113% at 4, 0, and −4°C compared to CK, respectively (Figure 2C). Whereas, under CS conditions, the SP contents of seedlings with Tre application were significantly increased by 134, 142, and 131% at 4, 0, and −4°C compared to CK, respectively (Figure 2C). In short, the CS reduces the SS and SP contents, and exogenous Tre significantly increased the Pro, SS, and SP contents at CK and CS conditions.

#### Tre-Induced Malondialdehyde and H_2_O_2_ Levels in Cold-Stressed Rapeseed Seedlings

**FIGURE 3:**
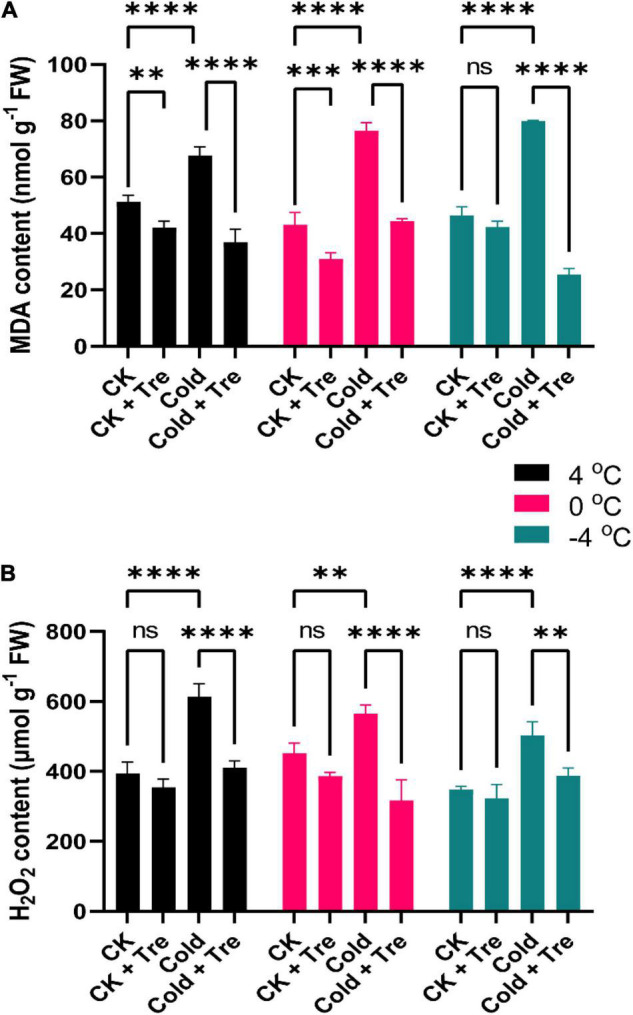
Impact of Tre on the levels of malondialdehyde (MDA) and hydrogen peroxide (H_2_O_2_) in rapeseed seedling under cold stress conditions. **(A)** MDA contents, and **(B)** H_2_O_2_ contents under four treatments [control (CK), CK + Tre, Cold, and Cold + Tre] and three stress conditions (4, 0, and –4°C). Tre indicates the application of 20 mM L^– 1^ of Tre. Data are expressed as the mean (± SD) from three biological replications for each sample and treatment. Asterisks show significant levels at ^****^P < 0.0001, ^***^P ≤ 0.001, ^**^P ≤ 0.01, and ns means non-significant.

Under CS conditions, the MDA level was increased by 132, 177, and 172% at 4, 0, and −4°C compared to CK, respectively (Figure 3A). The CS also resulted in increased H_2_O_2_ levels by 155, 125, and 144% at 4, 0, and −4°C compared to CK, respectively (Figure 3B). Under normal conditions, the exogenous Tre significantly reduced the MDA contents by 82, 72, and 91% at 4, 0, and −4°C, compared to CK, respectively (Figure 3A). Likewise, the exogenous Tre application also reduced the H_2_O_2_ levels by 90, 85, and 92% at 4, 0, and −4°C compared to CK, respectively (Figure 3B). Whereas under CS conditions, the MDA content was declined significantly by 72% at 4°C, and 54% at −4°C compared to CK, respectively (Figure 3A). Under CS conditions (4 and −4°C), there was no considerable difference in the H_2_O_2_ levels compared to CK (Figure 3B). Notably, the H_2_O_2_ level was only reduced at 0°C (by 70%) than the CK (Figure 3B). Briefly, the exogenous Tre substantially reduced the MDA and H_2_O_2_ levels in cold-stressed and CK seedlings compared to CK.

#### Exogenous Tre Regulates the Antioxidant Enzyme Activities in Cold-Stressed Rapeseed Seedlings

**FIGURE 4:**
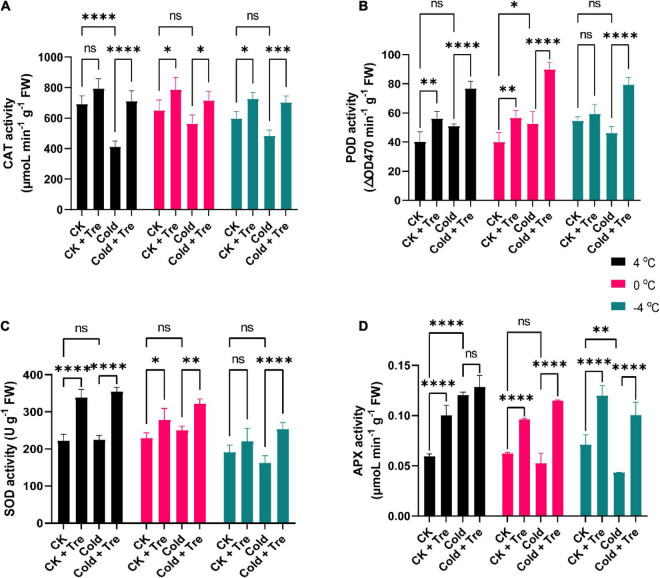
Impact of Tre on antioxidant enzyme activities in rapeseed seedling under cold stress conditions. **(A)** Catalase (CAT) activity, **(B)** peroxidase (POD) activity, **(C)** superoxide dismutase (SOD) activity, and **(D)** ascorbate peroxidase (APX) activity under four treatments [control (CK), CK + Tre, Cold, and Cold + Tre] and three stress conditions (4, 0, and –4°C). Tre indicates the application of 20 mM L^– 1^ of Tre. Data are expressed as the mean (± SD) from three biological replications for each sample and treatment. Asterisks show significant levels at ^****^P < 0.0001, ^***^P ≤ 0.001, ^**^P ≤ 0.01, *P ≤ 0.05, and ns means non-significant.

Cold stress substantially increases the ROS production in plants compared to the CK plants. Thus, the improved activities of antioxidant enzymes, including CAT, POD, SOD, and APX, adjust the equilibrium of ROS production under CS conditions. In this research, the CS reduced the CAT activity by 59, 86, 80% at 4, 0, and −4°C compared to CK, respectively (Figure 4A). Under normal conditions, the exogenous Tre significantly increased the CAT activity by 114, 120, and 121% at 4, 0, and −4°C of rapeseed seedlings treated with Tre. Likewise, under CS conditions, the CAT activity was boosted by 102, 110, 117% at 4, 0, and −4°C compared to CK, respectively (Figure 4A). Cold increases the POD activity by 126% at 4°C, and 131% at 0°C compared to CK (Figure 4B). However, under normal conditions, the exogenous Tre significantly increased the POD activity by 139, 141, and 108% at 4, 0, and −4°C compared to CK, respectively (Figure 4B). Under CS conditions, the POD activity was substantially augmented by 190, 225, and 145% at 4, 0, and −4°C compared to CK, respectively (Figure 4B).

Under CS conditions (mainly at −4°C), the SOD activity was reduced by 84% compared to CK, whereas it was slightly improved at 4 and 0°C conditions (Figure 4C). The exogenous Tre increases the SOD activity by 152, 121, and 115% in CK seedlings with Tre application at 4, 0, and −4°C compared to CK, respectively (Figure 4C). Under CS conditions, the SOD activity was increased by 160, 140, and 132% at 4, 0, and −4°C compared to CK, respectively (Figure 4C). Under CS conditions, mainly at 0°C, and −4°C, the APX activity was substantially dropped by 84 and 61% compared to CK, respectively, whereas it was significantly increased by 202% at 4°C (Figure 4D). However, under normal conditions, the Tre application enhanced the APX activity by 168, 155, and 168% at 4, 0, and −4°C compared to CK, respectively (Figure 4D). Under CS conditions, the APX activity was significantly boosted by 216, 184, and 141% at 4, 0, and −4°C compared to CK, respectively (Figure 4D). Overall, our results showed that CS alone reduced the antioxidant enzyme activities. Therefore, the exogenous application of Tre remarkably increased the CAT, POD, SOD, and APX activities to cold-stressed and CK seedlings with the application of Tre. The highest activities were recorded with the Tre treatment throughout the CS conditions.

### Impact of Tre on the Cold Stress-Responsive Marker and Tre-Biosynthesis Genes in Cold-Stressed Rapeseed Seedlings

#### Effect of Tre on the Expression Levels of Antioxidant Genes

**FIGURE 5:**
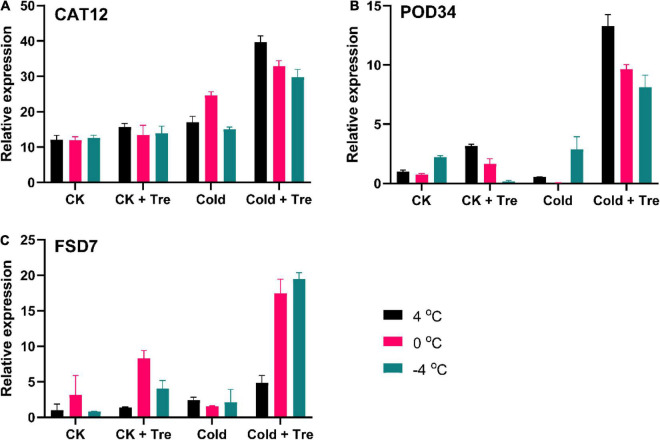
Impact of Tre on the relative expression of antioxidant enzyme-encoding genes in rapeseed seedling under cold stress conditions. **(A)** the relative expression of catalase (*CAT12*) gene, **(B)** the relative expression of peroxidase (*POD34*) gene, and **(C)** the relative expression of superoxide dismutase (Fe-SOD/*FSD7*) gene under four treatments [control (CK), CK + Tre, Cold, and Cold + Tre] and three stress conditions (4, 0, and –4°C). Data are expressed as the mean (± SD) from three technical replications for each sample and treatment.

In plants, the ROS production is mainly regulated by an antioxidant defense system (Hasanuzzaman et al., 2020). In this study, the exogenous Tre significantly increased the antioxidant enzyme activities (Figure 4). Therefore, we also evaluated the impact of Tre on the expression level of antioxidant-encoding genes (Figure 5). The exogenous Tre significantly increases the expression level of the *CAT12* gene by 328, 275, and 235% at 4, 0, and −4°C compared to CK, respectively (Figure 5A). Under normal conditions, the Tre also slightly increases the *CAT12* expression (Figure 5A). Likewise, the *POD34* expression level was increased by 316% at 4°C, and 217% at 0°C in the seedlings treated with Tre compared to CK under normal conditions (Figure 5B). In cold-stressed seedlings, the *POD34* expression level of seedlings with Tre application was significantly boosted at 4, 0, and −4°C compared to CK, respectively (Figure 5B). Under CS conditions, the expression level of the Fe-SOD gene (*FSD7*) was dramatically increased at 4, 0, and −4°C compared to CK, respectively (Figure 5C). The maximum expression levels of *CAT12, POD34*, and *FSD7* were recorded in cold-stressed seedlings when treated with Tre (Figure 5).

#### Effect of Tre on the Expression Levels of Cold-Related Marker Genes

**FIGURE 6:**
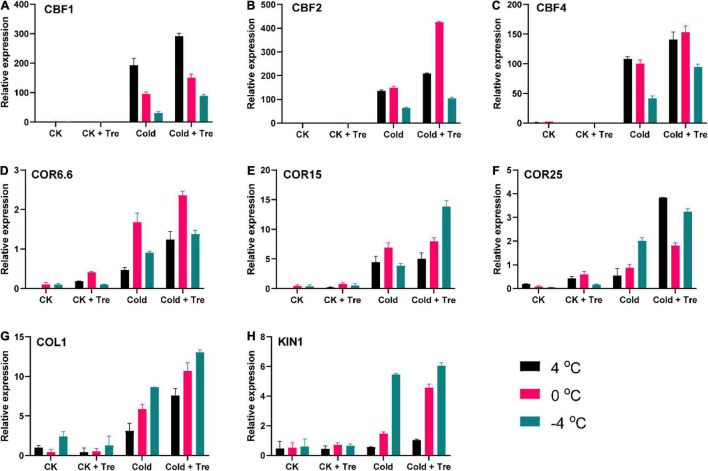
Impact of Tre on the relative expression of cold stress-related marker genes in rapeseed seedling under cold stress conditions. **(A–C)** the relative expression of C-repeat/dehydration responsive element (CRT/DRE) binding factor (*CBF1, CBF2*, and *CBF4*) genes; **(D–F)** the relative expression of cold-regulated (*COR6.6, COR15*, and *COR25*) genes; **(G)** the relative expression of Collagen 1 (*COL1*) gene, and **(H)** the relative expression of *KIN1* gene under four treatments [control (CK), CK + Tre, Cold, and Cold + Tre] and three stress conditions (4, 0, and –4°C). Data are expressed as the mean (± SD) from three technical replications for each sample and treatment.

The *CBF* and *COR* genes participate in CS signaling in plants. Earlier, it was described that the promoter sections of *COR6.6*, *COR15*, and *COR25* genes comprise C-repeat/dehydration responsive element (CRT/DRE), which could be bound by *CBF* genes (Thomashow, 1999). To get further insights on the Tre-induced regulation of gene expressions, we observed the expression levels of *CBF* and *COR* genes (Figure 6). In cold-stressed seedlings, the exogenous Tre significantly induced the expressions of *CBF1*, *CBF2*, and *CBF4* at 4, 0, and −4°C compared to CK, respectively (Figures 6A–C). Likewise, the expressions of *COR6.6*, *COR15*, and *COR25* were substantially enhanced with Tre treatment in cold-stressed seedlings at 4, 0, and −4°C compared to CK, respectively (Figures 6D–F). Whereas at normal conditions, the expression levels of *COR6.6* and *COR25* were slightly induced by exogenous Tre than CK (Figures 6D,F). However, there was no considerable change in the *COL1* and *KIN1* expression levels under normal conditions with or without exogenous Tre. However, under CS conditions, the exogenous Tre remarkably boosted the expression levels of *COL1* and *KIN1* at 4, 0, and −4°C compared to CK, respectively (Figures 6G,H). Collectively, all the cold-related genes showed higher expression levels in the seedlings treated with exogenous Tre under CS conditions.

#### Effect of Tre on the Expression Levels of Tre-Biosynthesis Genes

**FIGURE 7:**
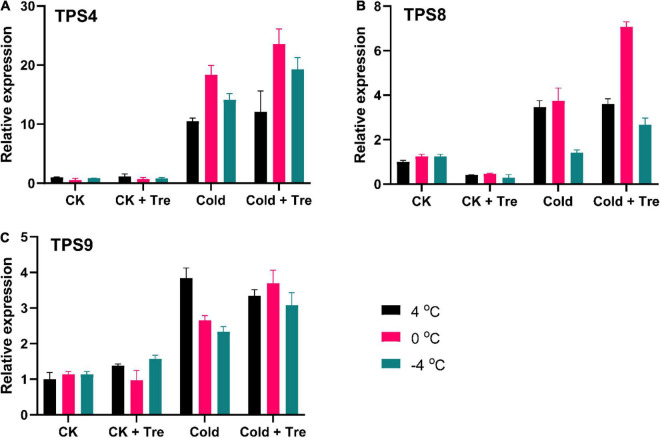
Impact of Tre on the relative expression of trehalose-6-phosphate synthase (TPS)-biosynthesis genes in rapeseed seedling under cold stress conditions. **(A)** the relative expression of *TPS4* gene, **(B)** the relative expression of *TPS8* gene, and **(C)** the relative expression of *TPS9* gene under four treatments [control (CK), CK + Tre, Cold, and Cold + Tre] and three stress conditions (4, 0, and –4°C). Data are expressed as the mean (± SD) from three technical replications for each sample and treatment.

To disclose further insights into the influence of Tre on the regulation of Tre-biosynthesis genes, we evaluated the expressions of Tre-biosynthesis genes in cold-stressed seedlings (Figure 7). The outcomes exhibited that the exogenous Tre significantly augmented the expression levels of *TPS4*, *TPS8*, and *TPS9* genes in the cold-stressed seedlings at 4, 0, and −4°C compared to CK, respectively (Figures 7A–C). With the application of Tre under normal conditions, there was no substantial change in the expression of the *TPS4* gene, but the expression of *TPS8* was reduced. On the other hand, the expression of *TPS9* was slightly increased by 138% at 4 and −4°C compared to CK (Figures 7A–C). The results showed that *TPS4, TPS8*, and *TPS9* also responded to CS greatly; however, the Tre treatment significantly boosted their expression levels in cold-stressed seedlings.

### Correlation Analysis

**FIGURE 8:**
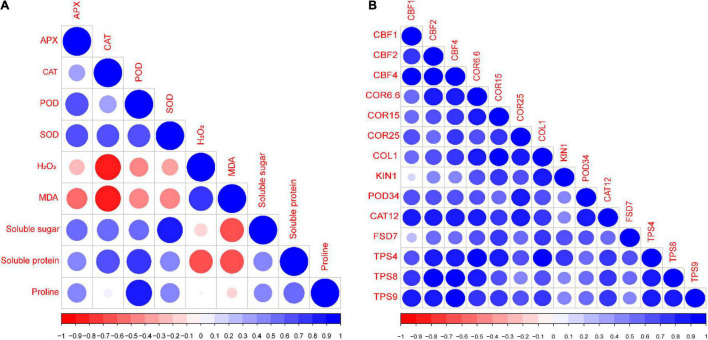
Pearson’s correlation analysis between **(A)** different studied indices and **(B)** stress-responsive genes of rapeseed seedlings grown under four treatments [control (CK), CK + Tre, Cold, and Cold + Tre] and three stress conditions (4, 0, and –4°C). Blue and red colors indicate positive and negative correlation, respectively.

A Pearson’s correlation analysis was performed among different evaluated indices and genes of the rapeseed seedlings (Figure 8). The correlation analysis showed that SS, SP, and Pro were positively correlated with APX, CAT, POD, and SOD; meanwhile, it was negatively correlated with H_2_O_2_ and MDA (Figure 8A). Similarly, all the antioxidants (APX, CAT, POD, and SOD) were positively correlated with each other. Additionally, H_2_O_2_ and MDA were negatively correlated with APX, CAT, POD, and SOD (Figure 8A). Likewise, the gene correlation analysis showed that all the genes were strongly and positively correlated with each other (Figure 8B). This correlation represents a close relationship between osmoprotectants, antioxidants, stress-responsive genes, and Tre-induced CS tolerance in the rapeseed seedlings.

### Principal Component Analysis

**FIGURE 9:**
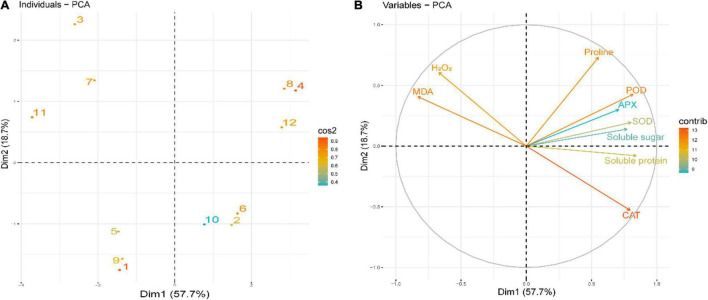
Principal component analysis (PCA) of **(A)** individual treatments by PCA and **(B)** different studied indices of rapeseed seedlings grown under four treatments [control (CK), CK + Tre, Cold, and Cold + Tre] and three stress conditions (4, 0, and –4°C). **(A)** Score plot signifies the partition of treatments as (1) CK_4°C, (2) CK + Tre_4°C, (3) Cold_4°C, (4) Cold + Tre_4°C, (5) CK_0°C, (6) CK + Tre_0°C, (7) Cold_0°C, (8), Cold + Tre_0°C, (9) CK_–4°C, (10) CK + Tre_–4°C, (11), Cold_–4°C, and (12) Cold + Tre_–4°C. Other abbreviations are defined in the main text.

To evaluate the effect of Tre treatments on the studied indices of rapeseed seedlings, the score and loading graphs of PCA were executed (Figure 9). The first two components, such as Dim1 (PC1, 57.7%) and Dim2 (PC2, 18.7%) showed the greatest involvement and presented 76.4% of the total variance in the dataset. Concerning CS conditions, the same treatments at different temperatures were clustered nearby, whereas the different treatments were separated effectively by the first two components (Figure 9A). This separation of the treatments clearly indicated that the Tre treatment under CS had a substantial ameliorative impact on the studied indices of rapeseed seedlings compared to CK. The first group of the variables of PCA, i.e., PC1, is positively correlated, involving the variables like CAT, SOD, POD, APX, SP, SS, and Pro (Figure 9B). On the contrary, a significant negative correlation of PC1 variables involves H_2_O_2_ and MDA that are aligned with PC2 (Figure 9B).

## Discussion

Rapeseed is a vital oilseed crop worldwide; however, CS significantly affects its growth and production (He et al., 2021; Mehmood et al., 2021; Raza, 2021; Raza et al., 2021e; Su et al., 2021b). Exogenously applied plant growth regulators (phytohormones, osmolytes, neurotransmitters, etc.) can successfully improve the CS tolerance in different crop plants (Diao et al., 2017; Hu et al., 2017; Waqas et al., 2017; He et al., 2021). Among them, Tre is considered as the “sugar of life” due to its phenomenal protective role against numerous abiotic factors, including high salinity, drought, osmotic pressure, waterlogging, and extreme temperature (Kosar et al., 2019). However, the effect of exogenous Tre in improving the CS tolerance in the rapeseed is still uncertain. Therefore, in the present study, exogenous Tre (20 mM L^–1^) significantly improved the survival rate of cold-stressed rapeseed seedlings (Figure 1). Cold stress alone reduces the growth of the seedlings, while the Tre application showed a protective role. These results are consistent with the previous findings, such as cold-stressed tomato plants showing improved survival rate and growth attributes with 10 mM of Tre treatment (Liu et al., 2020). In another study, seed priming with Tre and Tre + spermidine significantly improved the seed vigor and the growth of the cold-stressed rice seedlings (Fu et al., 2020). In wheat, exogenous Tre inhibited floret degeneration and improved the floret fertility in the apical spikelets, thereby considerably lowering the grain number per spike under CS conditions (Liang et al., 2021). The report in the rapeseed also supports the protective role of Tre in advancing the plant growth and production under adverse environmental conditions, mainly under CS conditions.

Previous reports show a positive association between osmoprotectants and CS tolerance in plants (Fu et al., 2020; Liu et al., 2020; He et al., 2021). Shifting the osmotic stability is meant to be adequate to retain the veracity and strength of the cell membrane of the plants to adapt to stress conditions. Proline has important roles in osmotic alteration in the stress signal transduction, and it also functions as an antioxidant. The upsurge of the Pro level under various stresses was reported earlier (Kaur and Asthir, 2015; Fu et al., 2020). In this study, the Tre treatment increases the SP, SS, and Pro contents in cold-stressed seedlings than CK and improves the CS tolerance (Figure 2). These findings are in agreement with the previous results of Fu et al. (2020), who found that Tre-primed seedlings significantly increase the SS and Pro contents in rice under CS conditions. Drought-stressed *Alpinia zerumbet* plants also showed an increase in leaf-free Pro contents with the foliar application of Tre (Zulfiqar et al., 2021a). In another study, Xie et al. (2015a) reported that exogenous Tre increased the SS and SP contents in wheat plants and improved the CS tolerance in wheat. Under salt stress, the exogenously applied Tre also upsurges the SS contents in *Arabidopsis* plants (Yang et al., 2014). In a recent report, exogenous Tre improves the oxidative damage triggered by high temperature, coordinating the influence of wheat on heat stress by regulating the gene expression (Luo et al., 2021). These findings indicate that exogenous Tre plays a significant part in regulating the osmotic substances because Tre participates in sugar metabolism mainly under stress conditions which are important to minimize the adverse effect of multiple stresses.

Induction of oxidative stress is a common consequence of abiotic factors (Hasanuzzaman et al., 2020), and CS triggers the ROS overproduction and imbalanced the redox state (Mittler, 2017; Ding et al., 2019; Hasanuzzaman et al., 2020). Nonetheless, ROS are vital for several essential natural progressions, such as cellular proliferation and dierentiation (Mittler, 2017). The MDA is interpreted as an end product of lipid peroxidation, and the regulated MDA contents disclose the oxidative stress levels in the plants (Gaweł et al., 2004), whereas H_2_O_2_ is a vital signaling molecule in regulating the stress responses in plants (Neill et al., 2002). In this study, the CS increased the H_2_O_2_ and MDA contents by decreasing the temperature (from 4*^o^*C to −4*^o^*C). Interestingly, the Tre treatment decreased the MDA contents mainly at 4*^o^*C and −4*^o^*C, and decreased the H_2_O_2_ contents at 0*^o^*C in cold-stressed rapeseed seedlings (Figure 3). However, alleviated or nearly no change in H_2_O_2_ contents at 4*^o^* and −4*^o^*C in the cold-stressed rapeseed seedlings was observed, indicating that Tre may have complex effects on the stressed rapeseed (Figure 3). It has been known that the H_2_O_2_ and MDA contents were closely associated with the extent of CS, which act as a negative symbol of the CS tolerance in the rapeseed (Yan et al., 2019; He et al., 2021), and H_2_O_2_ also serves as a signaling component. Our results are in agreement with the previous study (Liu et al., 2020), which also found that CS increased the MDA contents, and Tre treatment reduces the MDA contents in cold-stressed tomato plants. Similarly, Tre treatment reduces the level of MDA and thus improves the CS tolerance in wheat plants (Xie et al., 2015a). Under drought stress, exogenous Tre reduces the H_2_O_2_ and MDA contents in sweet basil (*Ocimum basilicum* L.) and improves drought tolerance by mitigating the adverse effect of oxidative stress (Zulfiqar et al., 2021b). Under CS conditions, the H_2_O_2_ contents were reduced over time, whereas the Tre treatment increased it in the tomato plants (Liu et al., 2020). At certain time points, there was no considerable influence on the H_2_O_2_ contents in cold-stressed plants (Liu et al., 2020). These results support our findings as there was no major divergence in the H_2_O_2_ contents at 4*^o^* and −4*^o^*C. In a recent report, exogenous Tre induces the rise in H_2_O_2_ and nitric oxide levels in cold stressed melon plants (Liu et al., 2021). In plants, CS leads to ROS production, and antioxidative enzymes display crucial responsibilities in preserving the redox stability by ROS scavenging (Mittler, 2017; Hasanuzzaman et al., 2020). The enhanced activities of POD, CAT, SOD, and APX are generally considered as markers of CS tolerance in plants (Yan et al., 2019; Liu et al., 2020; He et al., 2021). In the current study, the SOD, CAT, POD, and APX activities were significantly decreased under CS conditions, except on a few occasions, such as SOD had no significant change at 4 and 0°C; POD activity was increased at 4 and 0°C in cold-stressed seedlings (Figure 4). The cold-stressed melon plants increase the SOD, APX, and glutathione reductase activities with Tre application compared to CK (Liu et al., 2021). Interestingly, the Tre treatment significantly improved the activities of CAT, SOD, POD, and APX in cold-stressed rapeseed seedlings and improved the freezing tolerance (Figure 4). These results are in agreement with the previous studies (Xie et al., 2015a; Liu et al., 2020). Foliar application of Tre increases the SOD, POD, and CAT activities in drought stressed-sweet basil and improves the overall growth and physiological attributes (Zulfiqar et al., 2021b). Previous studies have shown that Tre can lessen ROS production in plants under abiotic conditions (temperature, drought, salinity, and heavy metals) by improving the antioxidant defense systems (Kosar et al., 2020). It was further confirmed by the correlation analysis where SP, SS, and Pro were positively correlated with SOD, POD, CAT, and APX; meanwhile, they were negatively correlated with the H_2_O_2_ and MDA contents (Figure 8). These observations proved that Tre also serves as a booster for improving the defense mechanisms in plants, mainly under adverse abiotic stress conditions.

As antioxidant enzyme plays a substantial part in ROS scavenging and improving the CS tolerance with the help of exogenous Tre, we evaluated the expression levels of antioxidant genes (Figure 5). In this study, it was observed that the CS induced the activities of antioxidant defense systems and the expression level of the relative genes. On the contrary, the exogenous Tre increases the expression levels of *CAT12, POD34*, and *FSD7* genes in the cold-stressed seedlings (Figure 5), indicating that SOD catalyzed oxygen change into H_2_O_2_, which was then switched to water/oxygen through CAT/SOD in cold-stressed seedlings (Mittler, 2002). This could facilitate the CS response in rapeseed and eventually lead to CS tolerance (Yan et al., 2019; Liu et al., 2020; He et al., 2021). It can be concluded that the CS induced the activities of antioxidants and cannot entirely negotiate additional ROS production. The exogenous Tre boosts the expression levels of antioxidant enzyme-encoding genes in the rapeseed seedlings. Similar observations have also been reported by Liu et al. (2020), who reported the increased expressions of *Cu/Zn-SOD, APX5, CAT1*, and *GR1* genes with Tre treatments in the cold stressed tomato leaves.

Until now, ICE-CBF-COR signaling pathways have been clearly characterized under CS conditions (Wang et al., 2017; Shi et al., 2018; He et al., 2021). Thus, the expression levels of CS-responsive marker genes, including *CBFs, CORs, COL1*, and *KIN1*, were evaluated (Figure 6). The results showed that Tre treatment substantially enhanced the expression levels of *CBF1, CBF2, CBF4, COR6.6, COR15, COR25, COL1*, and *KIN1* genes in cold-stressed rapeseed seedlings compared to CK (Figure 6). It signified that Tre was involved in the transcriptional regulation, mainly by altering the expression levels of *CBFs* and *CORs* genes. Recently, it was described that the exogenous treatment with other plant growth regulators like melatonin improves the CS tolerance by adjusting the expression levels of *AtCBF1-AtCBF3* and *COR15a* genes in *Arabidopsis* (Shi et al., 2015). Similar results were recorded in the rapeseed plants under CS, where melatonin increases the expression levels of several CS-responsive marker genes, including *CBFs, CORs, COL1*, etc., (He et al., 2021). In the near future, more insightful studies are required to fully explore the Tre-induced increase in gene expression levels, mainly under CS conditions.

Further, the expression levels of Tre-biosynthesis genes were also evaluated (Figure 7). Recent findings showed that the *TPS* gene family mainly responds to several abiotic factors in plants (Yang et al., 2012; Xie et al., 2015b; Rahman et al., 2021). In the present study, we observed that *TPS4, TPS8*, and *TPS9* significantly responded to CS, whereas their expression levels increased with Tre treatments in cold-stressed rapeseed seedlings (Figure 7). The highest expression levels were detected at 0°C with Tre treatment. In a recent study, it was observed that the Tre treatments increased the expression levels of *OsTPP1* and *OsTPP2* genes in rice plants under CS conditions (Fu et al., 2020). Further, Joshi et al. (2020) reported that the increased Tre biosynthesis improves yield potential in rice plants under drought, saline, and sodic conditions. It was further confirmed by correlation analysis, where all the expression levels of investigated genes were positively correlated with each other (Figure 8) which helped the rapeseed plants to survive under CS conditions. Similarly, the overexpression of *TPS* genes also increased the endogenous Tre contents and improved the tolerance to multiple abiotic stress conditions, such as salinity (Ge et al., 2008; Lyu et al., 2013), cold (Liu et al., 2019), heat (Lyu et al., 2018), drought (Suárez et al., 2008; Lyu et al., 2013; Nuccio et al., 2015; Lin et al., 2019), and high light (Rathod et al., 2016). Therefore, we proposed that the high expression levels of Tre-biosynthesis genes could increase the endogenous Tre contents and stress tolerance.

## Conclusion

**FIGURE 10:**
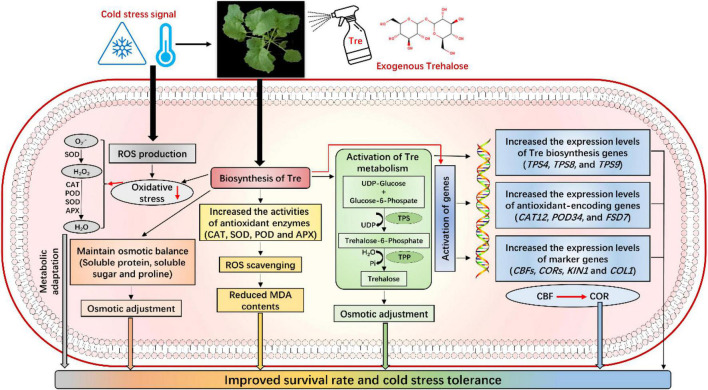
Mechanism of Tre-induced cold stress tolerance in rapeseed seedlings. Exogenous Tre not only regulates the antioxidant defense system and osmotic adjustment, but it also significantly participates in the activation of Tre metabolism and signaling network to improve cold tolerance in rapeseed by regulating the expression levels of stress-responsive genes.

In this study, exogenous Tre improved the CS tolerance in the rapeseed seedlings by maintaining the contents of osmoprotectants. Under CS conditions, Tre treatments also activate the antioxidant defense systems and the expression levels of antioxidant-encoding genes to cope with ROS production, thus eventually reducing the MDA damage and lessening the CS-induced growth inhibition. Additionally, Tre also contributed to the signaling network of CS tolerance by increasing the expression levels of markers genes (*CBFs, CORs, KIN1*, and *COL1*) and Tre-biosynthesis genes. It can be determined that the exogenous Tre not only regulates the antioxidant and osmotic balance but also significantly participates in sugar and Tre metabolism to improve CS tolerance in plants (Figure 10). Thus, the current study boosted our understanding of Tre-mediated CS response and tolerance in the rapeseed seedlings. In the near future, more in-depth studies are required to fully uncover the Tre-induced gene expression, leading to increased endogenous Tre contents and improving the stress tolerance under various stress conditions.

## Data Availability Statement

The original contributions presented in the study are included in the article/supplementary material, further inquiries can be directed to the corresponding author/s.

## Author Contributions

AR conceived the idea and wrote the manuscript. AR and WS performed the experiment. WS, ZJ, DL, YZ, AG, MH, and SM helped in literature search and data analysis. YC, XZ, and YL supervised the work, reviewed, and edited the manuscript. All authors have read and approved the final version of the manuscript.

## Conflict of Interest

The authors declare that the research was conducted in the absence of any commercial or financial relationships that could be construed as a potential conflict of interest.

## Publisher’s Note

All claims expressed in this article are solely those of the authors and do not necessarily represent those of their affiliated organizations, or those of the publisher, the editors and the reviewers. Any product that may be evaluated in this article, or claim that may be made by its manufacturer, is not guaranteed or endorsed by the publisher.

## Footnotes

1
http://www.geruisi-bio.com/pro

2
http://cbi.hzau.edu.cn/bnapus/index.php
